# Comparison of Bone‐setting Robots and Conventional Reduction in the Treatment of Intertrochanteric Fracture: A Retrospective Study

**DOI:** 10.1111/os.13954

**Published:** 2023-12-12

**Authors:** Zhanmin Xu, Xinan Zhang, Yongqing Wang, Xiaohui Hao, Meiyue Liu, Jingtao Sun, ZhiHui Zhao

**Affiliations:** ^1^ Tianjin Fourth Centre Hospital Tianjin China; ^2^ Tianjin University of Traditional Chinese Medicine Tianjin China

**Keywords:** Bone setting, Conventional reduction, Femur intertrochanteric fracture, Robot

## Abstract

**Objective:**

Intertrochanteric fracture of the femur is a common fracture in older people. Due to the poor systemic condition and prognosis of elderly patients, it is prone to more complications. We introduce the bone‐setting concept in the design of the robots, which are used for intertrochanteric fracture of the femur reduction. The purpose of this study is to compare the effect of bone‐setting robots and conventional reduction in the treatment of intertrochanteric fracture of the femur (IFF).

**Methods:**

From June 2021 to January 2023, 60 patients with IFF who were treated surgically were assigned to bone‐setting robots group and conventional reduction methods group in this retrospective study. The reduction time, operation time, total time, intraoperative blood loss, incision length, fluoroscopy time, and the follow‐up time were reviewed. The visual analogue scale (VAS) and Harris scores were used for functional assessment. For continuous variables, independent *t*‐tests were applied; for categorical data, the chi‐square test was applied. The significance level as *p* < 0.05.

**Results:**

Among the 60 patients with IFF, 31 were assigned to the bone‐setting robots group, and 29 were assigned to the conventional reduction methods group. Both groups with a similar baseline in the number, gender, age, and classification (*p* > 0.05). The reduction time, operation time, total time, intraoperative blood loss, and fluoroscopy time were less than those in the bone‐setting robots reduction group compared to the conventional reduction group. In the bone‐setting robots reduction group, the preoperative VAS score was 6.2 ± 1.3, the Harris score was 35.3 ± 3.1, 1 week after surgery VAS score was 3.3 ± 1.2, the Harris score was 57.3 ± 3.7, and at the last follow‐up VAS score was 2.4 ± 0.8, and the Harris score was 88.7 ± 3.4. While in the conventional reduction group, the preoperative VAS score was 6.3 ± 1.3, the Harris score was 35.9 ± 2.9, 1 week after surgery VAS score was 4.8 ± 1.4, the Harris score was 46.8 ± 2.8, and at the last follow‐up VAS score was 2.6 ± 0.8, and the Harris score was 87.3 ± 3.3. There were no significant differences between the two groups at the preoperative and 6‐month postoperative follow‐ups in VAS score and Harris score (*p* > 0.05, *p* > 0.05, respectively). But the difference was statistically significant at the one‐week postoperative follow‐up in VAS and Harris scores (*p* < 0.001).

**Conclusion:**

The bone‐setting robots can better protect the “fracture environment” and have the advantages of being precise, minimally invasive, simple, short time, low radiation, and rapid fracture recovery. The clinical effect of closed repair of IFF is ideal.

## Graphical abstract

The flow chart of surgery.

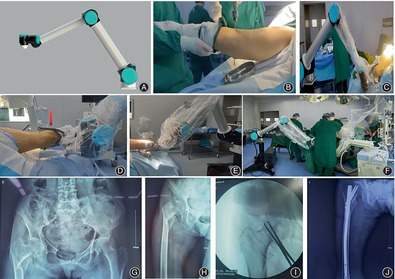

## Introduction

Intertrochanteric fracture of the femur (IFF) is a prevalent form of hip fracture in elderly populations, comprising 50% of all hip fractures and 23.79% of total body fractures.^1^ Conservative treatments may not be optimal, and patients may experience complications such as decubitus ulcers and pneumonic pneumonia. Consequently, surgical intervention is recommended for IFF in elderly patients.^2^ However, multiple surgical methods exist for treating IFF, among which the nail plate fixation device and the intramedullary fixation device are the two main techniques.^3^ Of these, intramedullary nailing of the proximal femur enjoys a reputation for being reliable, minimally invasive, centrally anatomical, and having low complication rates, thereby offering significant clinical utility.^4^, ^5^

Optimal fracture reduction is crucial for effective internal fixation treatment, regardless of the surgical technique. Traction bed reduction is the predominant approach used for intramedullary nail fixation.^6^ However, this approach has several limitations, including the prolonged installation time required for the traction bed, cumbersome operation, and often an unsatisfactory and challenging repositioning process. Moreover, patients undergoing traction bed must maintain a supine position, which can hinder sufficient internal retraction of the affected limb, leading to higher risks of contamination of the surgical incision and difficulty placing intramedullary nails due to the iliac wing obstruction. Additionally, the cross‐joint traction approach for multi‐angle traction reset is challenging in quantifying rehabilitation parameters.^7^, ^8^ An alternative approach involves continuous freehand traction, but this strategy cannot sustain the fracture reduction state in the long term, requires regular maintenance, and increases bone and soft tissue damage, as well as the operation time, bleeding, infection rate, and radiation exposure for both patients and medical staff.

Presently, robotic‐assisted surgery has become widely utilized for surgical positioning of the spine and joints in orthopedics.^9^, ^10^ However, due to the diversity and complexity of trauma surgery, the intricate and variegated nature of fracture types, and the array of internal fixation material options, current orthopedic robots cannot reliably reset and maintain the fracture reduction state and fixation procedures. The varying location of the fracture end further complicates effective internal fixation with robotic assistance.^11^, ^12^, ^13^ Currently, orthopedic robots are only used for pelvic fracture screw positioning and fixation, with no reported usage in clinical fracture reduction and fixation of extremities.

**FIGURE 1:**
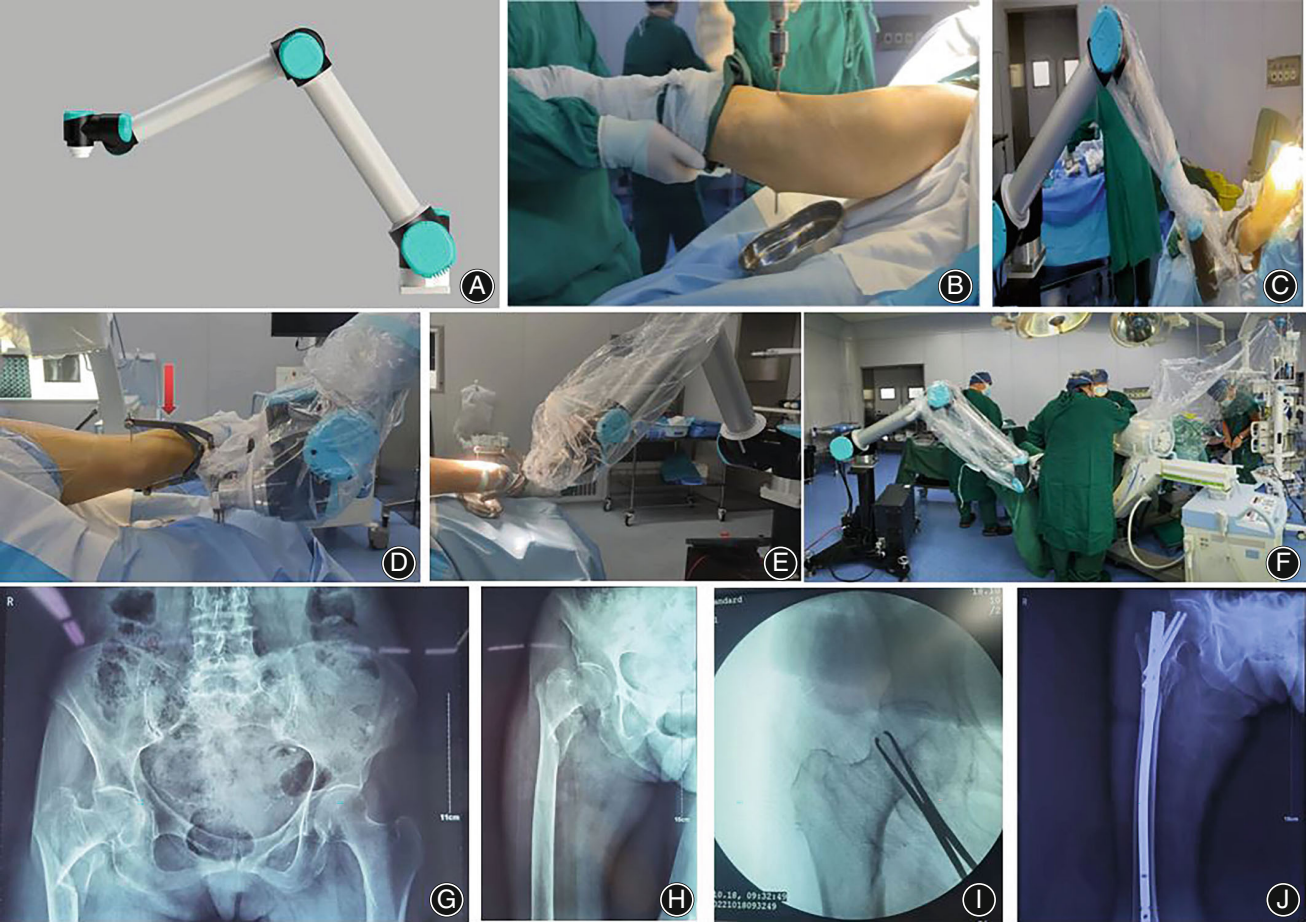
(A) Schematic picture of the bone‐setting robots arm. (B) A 3.0 mm Kirschner wire was inserted into the patient's femoral condyle from the outside along the direction of the intercondylar line. (C) The Kirschner wire was attached to the bone‐setting robot arm from the traction bow. (D) The Kirschner wire was attached to the bone‐setting robot arm from the traction bow (red arrow indicates the traction bow). (E) Fracture reduction was achieved by traction of the bone‐setting robots arm. (F) Panorama of bone‐setting robots operating room. (G) The preoperative x‐ray photographs of anteroposterior pelvis view showing the right‐side femoral intertrochanteric fracture. (H) The preoperative x‐ray photographs of anteroposterior proximal femur view showing the right‐side femoral intertrochanteric fracture. (I) The intraoperative C‐arm x‐ray photographs of anteroposterior proximal femur view showing the fracture was reduced. (J) The postoperative x‐ray photographs of anteroposterior proximal femur view showing the internal fixation of the fracture site.

Our orthopedic robots were purposefully designed with the bone‐setting concept in mind and jointly developed by hospitals and enterprises. The mechanical arm boasts seven degrees of freedom of movement, a minimum tensile force of 20 kg, and ensures sterility during fracture reduction procedures. By incorporating six critical revision action parameters, our robots effectively replace the traditional traction beds and enable closed revision of IFF. In this retrospective study, we conducted a comparative analysis by simultaneously evaluating traction bed resurfacing to assess the clinical outcomes of our bone‐setting robots in treating IFF (Figure 1). Our study aimed to achieve the following objectives: (i) evaluate the feasibility and benefits of utilizing bone‐setting robots to correct IFF; (ii) discuss operational points and precautions during robot‐assisted IFF treatment; and (iii) address limitations and outline future development directions of the bone‐setting robots.

## Materials and Methods

### General Information

All data of this study were approved by the ethics department of Tianjin Fourth Central Hospital (SZXLL‐2022‐H003), and all patients signed the informed consent form for surgery and ethical, informed consent. The AO classification of IFF was used in all of the patients. All patients took an x‐ray of the affected hip joint and 3D reconstruction of the hip CT and planned closed revision fracture and percutaneous minimally invasive intramedullary nail fixation according to the fracture morphology and staging.

### Inclusion and Exclusion Criteria

The inclusion criteria were as follows: (i) intertrochanteric fractures of the femur; (ii) AO/OTA fracture type 31‐A1, 31‐A2 fractures; (iii) the time between injury and surgery did not exceed 3 weeks; and (iv) internal fixation with intramedullary nailing with bone setting robotic assistance or convention reduction implantation methods.

The exclusion criteria were as follows: (i) old intertrochanteric fracture of the femur; (ii) severe osteoporosis, bone mineral density T value <3.0 measured by dual energy x‐ray absorptiometry (DXA); (iii) pre‐injury hip mobility impairment, deformity of femur development, and so forth, affecting the function of the affected limb; (iv) uneven or deformed pelvis; (v) combined ipsilateral femoral stem fractures were excluded; and (vi) insufficient follow‐up data, follow‐up time less than 6 months.

### Robot Introduction

The bone‐setting robots system was manufactured by Tianjin Yangtian Technology Co. Ltd (Tianjing, China; mainly responsible for the robotic arm production) and Jiasite Medical Equipment (Tianjin) Co. Ltd. ((Tianjing, China; mainly responsible for the robotic software development). The bone‐setting robots have been designed using a modular concept, with a hollow mechanical arm and an internal wiring structure. It features six joints, seven degrees of freedom, and a minimum force requirement of 20 kg, making it a new‐generation robot system. The joint movement range spans from ±180° to 360°, with a maximum joint speed of 120 mm/s to 180 mm/s and a repeat positioning accuracy within ±0.02 mm. The bone‐setting robots utilized a one‐key reduction program to activate the robotic arm, applying a traction force of 10.3 ± 2.6 kg (range 9.6–12.5 kg), abduction of 18.3° ± 4.6° (range 16°–21°), flexion of 20° ± 8.8° (range 18°–26°), and internal rotation of 45° ± 13.6° (range 36°–56°) to achieve fracture reduction.

Before the start of reduction, we placed a 3.0 Kirschner wire into the distal end of the femur. One end of the traction bow was connected to the Kirschner wire, and the other end was connected to the mechanical arm. The bone‐setting robots can perform manual and one‐button automatic reduction programs through the robotic software. During the reduction process, the robot arm automatically rotates and applies traction, exerting continuous and stable force to achieve fracture reduction. We have classified fracture displacement into six types of movements and rotations along the *X*, *Y*, and *Z* axes, and the bone‐setting robots have six reduction (flexion, extension, supination, pronation, abduction, adduction) quantification parameters to ensure highly accurate reduction. During our preliminary research, we first used manual reduction with the world coordinate system, combining the *X*, *Y*, and *Z* axes of the patient and robot coordinate systems. Using a force feedback device installed on the reduction arm, we translated the longitudinal traction force of 10–15 kg, abduction of 15°–20°, internal rotation of 45°, and flexion of 20° into machine language to manually operate the robot for reduction. Based on patient data collected in our previous and current research, we have developed an RL language editing system for the one‐button reduction of IFF in the orthopedic robot. The system has been pre‐programmed into the orthopedic robot host for later use. With the help of the RL language, the system can determine the patient's fracture type and select the appropriate one‐button reduction program. However, manual reduction may still be required for certain patients if limitations to matching success exist due to the limited patient data.

### Surgical Technique

#### Anesthesia, Disinfection, and Shop Towels

In this research, both patient groups underwent intravenous combined anesthesia and received routine disinfection of affected limbs using iodine swabs, followed by covering with sterile surgical sheets per standard protocol. The same surgical team performed all surgical procedures.

### Surgical Position

A robotic surgical instrument table was secured to the operating bed, and a mechanical arm was employed to achieve real‐time positioning of the patient's sacrococcygeal and pubic symphysis via a fixed column, enabling the patient to assume a lateral position on the robotic surgical table. Concurrently, a folded sterile surgical sheet was utilized to cover the inner side of the affected thigh and the IFF. Both ends of the sheet were tautly pulled and affixed to the fixed column of the robotic surgical table to counteract the proximal region of the fracture's pulling and support. For patients subject to supine‐position operation, the traction bed reduction group was implemented with the affected‐side ankle being fastened to the traction bed, allowing for longitudinal, adduction, and internal rotation traction of the affected limb to reduce the fracture. The fixed column of the traction bed was placed outside the genitals and on the inner side of the thigh to counteract the traction.

### Connect the Robotic Arm

Regarding robot placement and connection, the bone‐setting robots arm has an impressive range of movement. It is fixed to the distal end of the patient's foot, with the mechanical arm being safeguarded by a sterile plastic sheath. Using a world coordinate adjustment mode, the robot arm is positioned above the femur's lateral condyle, after which the operator deploys a 3.0 mm Kirschner wire in the intercondylar line direction of the femoral condyle and fixedly connects it to the sterile traction mechanical hand. The direction and positioning of the traction and reduction that the robot arm will carry out are adjusted, and preparations for fracture reduction are made before the process. Note that the traction bed can solely be positioned at the distal end of the patient's foot, and a sterile soft connection device is utilized to establish a connection between the traction bed and the patient's ankle.

### Fracture Reduction

The robot program was developed by inputting the average values of six reduction parameters for IFF from previous studies. By referencing the lower extremity's force line and simulating traditional Chinese medical techniques, the force and angles were quantified with a longitudinal traction force of 10–15 kg, abduction of 15°–20°, internal rotation of 45°, and a flexion angle of 20°. At this stage, the fracture is typically reduced. In cases where the reduction is not satisfactory, a combination of three repeated rotations and three longitudinal stretching sessions is performed to achieve an acceptable anatomical reduction of the fracture ends. The traction bed reduction group applies longitudinal traction to the affected limb at a force of 10–15 kg, abduction of 15°–20°, and internal rotation of 45° to realign the fracture ends. Both groups of fracture reductions are performed under the monitoring of a C‐arm X‐ray machine with multiple‐angle perspectives to ensure satisfactory fracture reduction. Both groups used the standard surgical method of proximal femoral intramedullary nails to fix fractures.

### Efficacy Evaluation Indicators

#### Evaluation Index

The evaluation indexes include reduction time, operation time, total time, follow‐up time, blood loss (suction canister + gauze [30 mL/piece]), incision length, number of fluoroscopic times, visual analogue scale (VAS) score, and Harris score for functional recovery of the hip joint.

### Evaluation of Effectiveness

Reduction time—the time from the installation of the bone‐setting robots (or traction bed) to the satisfaction of the reduction of the fracture. Operation time—the time from the incision of the skin to the suture of the skin. Total time—the time from entering the operating room to the end of leaving the operating room after anesthesia recovery.

### Visual Analogue Scale (VAS)

VAS is a method for evaluating the degree of pain.^14^ The basic method is to use a movable ruler about 10 cm long with 10 markings on one side and “0” and “10” at both ends, representing no pain and the most severe pain that is difficult to endure, respectively. Patients choose a score out of 11 according to their condition. The scoring criteria are from 0 to 10, with evaluations of pain severity made before surgery, 1 week after surgery, and 6 months after surgery.

### Harris Score

The Harris score is a set of numerical rating standards proposed by Harris for evaluating hip joint function.^15^ The evaluation contents include pain, function, joint mobility, and deformity, with a full score of 100. Scores of 90–100 are considered excellent, 80–89 are considered good, 70–79 are considered fair, and scores below 70 are considered poor. The evaluation of hip joint function is made before surgery, 1 week after surgery, and 6 months after surgery.

### Statistical Analysis

We conducted a statistical analysis using SPSS 19.0 software (SPSS, Chicago, IL, USA). Continuous data, such as age, reduction time, operation time, total time, blood loss, incision length, fluoroscopy times, VAS scores, Harris scores were subjected to normality tests and presented as means ± standard deviations. Paired sample *t*‐tests were used for group comparisons. For categorical data, such as gender, fracture classification, and side, frequencies and percentages were used, and the chi‐square test was applied for group comparisons. The significance level was set at 0.05 for a two‐tailed test.

## Results

### General Results

The study included a total of 60 patients. Among them, 31 patients were included in the bone‐setting robots group (11 males and 20 females), with an average age of (78.7 ± 9.3) years and a range of 58 to 94 years. In total there were 16 fractures on the left side, and 15 on the right side. The AO/OTA classification was 31‐A1 in 17 cases and 31‐A2 in 14 cases. The remaining 29 patients were in the conventional reduction group (12 males and 17 females), with an average age of 79.0 ± 7.3 years and a range of 51 to 90 years. Among these patients, 15 had fractures on the left side, and 14 had fractures on the right side. The AO/OTA classification was 31‐A1 in 17 cases and 31‐A2 in 12 cases. There were no statistically significant differences observed in terms of sex, age, side, and AO/OTA classification between the two groups of patients (*p* > 0.05, Table 1).

**TABLE 1:** Demographics of patients with IFF whose treatment with bone‐setting robots group and conventional reduction group.

Group	*N*	Gender (*n*)		Age (year)	Affected side (*n*)		Classification	
		Male	Female		left	right	A1	A2
Bone‐setting robots	31	11	20	78.7 ± 9.3	16	15	17	14
Conventional reduction	29	12	17	79.0 ± 7.3	15	14	17	12
*t*	‐	0.22		−0.124	<0.001		0.087	
*p*	‐	0.639		0.902	0.993		0.786	

### Intraoperative Results

In the bone‐setting robots group, the operation time and the total time were less than the conventional reduction group. The two groups showed statistically significant differences in operation time, total time, and bleeding amount (*p* < 0.05, Table 2). Meanwhile, the amount of blood loss and fluoroscopy times were lower in the bone‐setting robots group compared to that of the conventional reduction group. Statistically significant differences were noted between the two groups in terms of blood loss and fluoroscopy times (*p* < 0.05, Table 3).

**TABLE 2:** Comparison of time between bone‐setting robots group and conventional reduction group.

Group	Reduction time	Operation time	Total time
Bone‐setting robots	4.4 ± 2.2	28.9 ± 13.5	103.2 ± 12.4
Conventional reduction	9.1 ± 3.1	47.8 ± 13.4	120.4 ± 26.7
*t*	−6.596	−5.444	−3.250
*p*	<0.001	<0.001	0.002

**TABLE 3:** Comparison of clinical results between bone‐setting robots group and conventional reduction group.

Group	Blood loss	Incision length	Fluoroscopy times	Follow‐up time
Bone‐setting robots	79.6 ± 25.5	5.8 ± 1.2	10.1 ± 2.5	26.9 ± 1.4
Conventional reduction	110.3 ± 36.2	5.7 ± 1.4	14.5 ± 3.2	27.4 ± 1.6
*t*	−2.884	1.322	−5.936	−1.136
*p*	<0.001	0.469	<0.001	0.261

### Visual Analogue Scale (VAS)

There are no significant differences between the two groups before and 6 months after the surgery (*p* > 0.05). But the VAS scores in bone‐setting robots were significantly lower than in the conventional reduction group 1 week after the surgery (*p* < 0.001, Table 4).

**TABLE 4:** Comparison of VAS score between bone‐setting robots group and conventional reduction group.

Group	Pre‐VAS	1 week post‐VAS	6 months post‐VAS
Bone‐setting robots	6.2 ± 1.3	3.3 ± 1.2	2.4 ± 0.8
Conventional reduction	6.3 ± 1.3	4.8 ± 1.4	2.6 ± 0.8
*t*	−0.447	−0.439	−1.157
*p*	0.656	<0.001	0.252

Abbreviations: Pre‐, preoperative; Post‐, postoperative; VAS, Visual Analog Scale.

### Harris Score

The comparison between the two groups showed no significant differences before and 6 months after surgery (*p* > 0.05). But the Harris score in bone‐setting robots was significantly more than in the conventional reduction group at 1 week after the surgery (*p* < 0.001, Table 5).

**TABLE 5:** Comparison of Harris score between bone‐setting robots group and conventional reduction group.

Group	Pre‐Harris	1 week post‐Harris	6 months post‐Harris
Bone‐setting robots	35.3 ± 3.1	57.3 ± 3.7	88.7 ± 3.4
Conventional reduction	35.9 ± 2.9	46.8 ± 2.8	87.3 ± 3.3
*t*	−0.707	12.396	1.658
*p*	0.482	<0.001	0.103

Abbreviations: Pre‐, preoperative; Post‐, postoperative.

### Complications

There were no intraprocedural adverse events in both groups and our research observed no postoperative complications, such as superficial wound infections, nonunion, delayed union, and arthritis. Meanwhile, bone‐setting robots have also confirmed that they are safe and reliable during surgery.

## Discussion

Our research found that patients with intertrochanteric fracture of the femur with bone‐setting robots treatment had the advantages of being precise, minimally invasive, simple, short time, and low radiation compared with patients who received conventional reduction. Although there was no difference between the two groups in terms of VAS and Harris scores at 6 months after surgery, the VAS and Harris scores were significantly better in the bone‐setting robots group than that in the conventional reduction group. This confirmed that the bone‐setting robots treatment had the advantage of rapid fracture recovery.

### The Advantages and Feasibility of Bone Setting Robots to Repair IFF

The technology of bone‐setting manipulation includes massaging, stretching, holding, pressing, kneading, pushing, and pulling techniques, is unmatched by other treatment methods to reduce further injury and promote fracture healing speed, thus reducing many complications. When a fracture occurs, it creates a unique “fracture environment” that induces local modifications in biology, physics, chemistry, and molecular biology, among other factors.^16^ These alterations gradually diminish as the fracture undergoes the healing process. The development of robots in orthopedics is due to the similarity of closed reduction fracture methods, principles, and methods that promote rapid fracture healing. Closed reduction fractures can reduce secondary injuries to bone membranes, muscles, ligaments, nerves, blood vessels, skin, and subcutaneous tissues. Meanwhile, the “fracture environment” under closed reduction conditions is conducive to fracture healing.^17^ The protective “fracture environment” is the critical basis for blood circulation establishment and fracture healing. Furthermore, we believe that quantifying the scoring of the “fracture environment” after treatment serves as a means to objectively assess the effectiveness of fracture treatment.^18^ In addition, retention of the hematoma around the fracture, which often originates from the bone marrow cavity after fracture, contains many bone marrow matrix stem cells. Therefore, the fracture site's hematoma represents a rich bone growth source.^19^

When this technology is combined with modern AI robots for fracture reduction, it means a significant development and advancement in orthopedic techniques. Clinical research indicates that robotic‐assisted reduction can provide six quantifiable parameters for longitudinal, medial, internal rotation, flexion, repeated stretching, and internal/external rotation, three more than the parameters supplied by traditional traction bed reduction. Additionally, the robot‐assisted method allows for precise quantification of each parameter. Adding the forward flexion position of the thigh parameter effectively avoids the obstruction of the iliac wing to the insertion of intramedullary nails. Furthermore, including repeated stretching and internal/external rotation reduction quantification parameters could effectively centralize the hematoma at the fracture site to the surrounding area, further reducing the extent of the fracture. The soft connection used in the traction bed reduction group to fix the affected side ankle and perform cross‐joint traction is not easy to quantify reduction. In contrast, the complicated connection between the femoral condyles used in the robot reduction group effectively avoids the loss of traction force angles in the hip joint. Finally, the sterile closed reduction of fractures on the operation bed represents an improvement in fracture reduction techniques compared to the traditional traction bed reduction performed under the operating table.^20^ The process is characterized by its simple operation methods, reduced operative time, convenient intraoperative adjustment, precise quantification of reduction, and one‐button intelligent reduction feature.^21^, ^22^, ^23^

### The Key Points in the Treatment of IFF with Bone‐setting Robot

A range of advantages can be obtained by changing a patient's position from supine to lateral. A lateral position allows for opposing lateral and upward traction on an injured hip, resulting in improved fracture reduction and perineum protection from injury. Compared to a supine position, a lateral position reduces the risk of surgical site contamination and provides better surgical visibility.^24^ Additionally, the procedure is more convenient for the entire internal fixation process. With the affected hip joint facing upward, the wound is elevated above the heart, reducing intraoperative blood loss associated with changing positions. The surgeon can also stand during the operation, allowing easier coordination with the surgical assistant on the opposite side. The lateral position permits increased thigh flexion, which relaxes the hip joint, a beneficial feature for obese patients to avoid iliac wing obstruction and reduce the potential for ligament tension.^25^ Ultimately, this technique transforms the traditional sterile traction bed reduction under the operation table to an AI‐based sterile closed reduction on the operation bed, significantly reducing the injury, risk, cost, and workload for patients, physicians, and nurses.^26^

The bone‐setting robot's traction requires a robust opposing force during the operation. We have designed and developed our robotic surgical table installed on top of the existing operating table, and is rigidly attached to it while also having vital fixing flexibility. Currently, we use the C‐arm image inspection method to determine whether the IFF has been successfully reduced. During the bone‐setting reduction process, a powerful external force is needed to separate the fractured near and far ends while firmly and effectively fixing and opposing the traction force of the near end, which plays a crucial role in whether the fracture can be reduced. In our preliminary experiments, we found that if the sterile surgical drape was not tightly and firmly fixed on the anchor of the surgical table, it would not be able to effectively oppose the traction force, which could lead to ineffective fracture reduction and changes in patient posture, resulting in prolonged reduction time and surgery time.

### Deficiency and Prospect of Bone‐setting Robot

During the early stages of development, the application scope of bone‐setting robots is relatively restricted, and several limitations exist. The bone‐setting robots for limb fracture reduction represent a completely new technology that poses challenges such as operator unfamiliarity, poor human‐machine interaction experience, and changes in the direction of traction during reduction. In the future, we will continue to enhance the mechanical arm, employ serial and parallel connection mechanisms and improve the collection and organization of data. We will continuously optimize the orthopedic robot platform system by integrating the orthopedic robot itself, the 3D C‐arm, the fracture dual‐micron fixator, the robot surgical table, and the automatic nailing robot creating an autonomous research and development system for an efficient “placement position, traction, reduction, and operation robot working platform.” The bone‐setting robot research has laid a robust foundation for developing surgical robot platforms. The combination of minimally invasive and micro‐stress shielding fixation techniques presents the possibility of using the limb fracture reduction surgical robot. At the same time, we also hope to introduce the concept of “fracture microenvironment” into future orthopedic clinical treatment and assessment.

The present study includes several limitations. First, this was a retrospective study, so missing data can lead to bias. Second, the sample size included was relatively limited, and the patients were not randomly assigned to each group; this may have resulted in selection bias. Third, there was no further discussion on functional outcomes with different fracture classifications.

## Conclusion

This study demonstrates that both the bone‐setting robots and conventional reduction are appropriate for the treatment of intertrochanteric fracture of the femur. Although there was no difference in terms of VAS and Harris scores between the two groups at 6 months after surgery, the bone‐setting robots can better protect the “fracture environment” and have the advantages of being precise, minimally invasive, simple, short time, low radiation, and rapid fracture recovery. The clinical effect of closed repair of IFF is ideal.

## Author Contributions

All authors contributed to the study conception and design. Material preparation, data collection and analysis were performed by Xinan Zhang, Qingyong Wang, Xiaohui Hao, Meiyue Liu, Jingtao Sun, Zhihui Zhao. The first draft of the manuscript was written by Zhanmin Xu and all authors commented on previous versions of the manuscript. All authors read and approved the final manuscript.

## Conflict of Interest

The authors, their immediate families, and any research foundation with which they are affiliated did not receive any financial payments or other benefits from any commercial entity related to the subject of this article. On behalf of all authors, the corresponding author states that there is no conflict of interest.

## Ethics Statement

The study protocol was reviewed and approved by the Ethics Committee of Tianjin Fourth Central Hospital and they are consent to participate in this study. Project number: SZXLL‐2022‐H003.
